# Neuroimaging in the early diagnosis of neurodegenerative disease

**DOI:** 10.1186/2047-9158-1-5

**Published:** 2012-01-13

**Authors:** A Jon Stoessl

**Affiliations:** 1 Pacific Parkinson's Research Centre, University of British Columbia & Vancouver Coastal Health, 2221 Wesbrook Mall, Vancouver, BC, V6T 2B5, Canada

## Abstract

Functional imaging may be useful for both the early diagnosis as well as preclinical detection of neurodegenerative disease. Additionally, while structural imaging has traditionally been regarded as a tool to exclude alternate diagnoses, recent advances in magnetic resonance show promise for greater diagnostic specificity. The role of MR and radionuclide imaging in early diagnosis and preclinical detection of dementia and parkinsonism are reviewed here.

## Introduction

Neurodegenerative disorders, of which Alzheimer's (AD) and Parkinson's (PD) diseases are the most common, take an enormous toll on affected patients and their families. For example, a recent analysis indicates that dementia and PD combined affect more than 7.5 million Europeans, at an estimated annual cost of €120 billion [1] and incalculable suffering. While symptomatic therapies are currently available, they are at best imperfect (PD), at worst provide only modest benefit (dementia) and they do not have any convincing impact on the inexorable progression of the underlying disorder. Advances in basic neuroscience make it increasingly likely that disease modifying therapies will be developed but these are likely to have maximal impact if they are introduced early in the course of the illness. Early disease detection would permit the provision of such therapies to those most likely to benefit from them, at the time that they are most likely to be effective. Early diagnosis permits the identification of people appropriate for inclusion in clinical trials of novel therapies and the exclusion of those who are not. Finally, early diagnosis allows better prognostication and appropriate resource utilization.

A variety of neuroimaging techniques may be useful for the early diagnosis of neurodegenerative disorders, but one should first consider the goal. Early diagnosis may refer to the timely correct differentiation of a specific disease entity from other conditions that may mimic it in early stages, or it may refer to the early detection of central nervous system dysfunction, prior to the emergence of clinical symptoms. The latter application is more likely to be of interest in populations at increased risk of disease, and could be useful for identification of subjects to participate in trials of neuroprotective agents, or ultimately to try and halt disease progression once effective disease-modifying interventions have been identified.

## Early differential diagnosis

### Parkinsonism

In the case of PD, the two major diagnostic considerations are either conditions that produce tremor but are not associated with dopamine deficiency (i.e. essential tremor or dystonic tremor) or other conditions that result in an akinetic-rigid syndrome, such as multiple system atrophy (MSA) or progressive supranuclear palsy (PSP). Imaging with radiotracers that assess presynaptic dopamine function such as single photon emission computed tomography (SPECT) using the dopamine transporter (DAT) ligand [^123^I]FP-CIT (DaTscan) will reliably differentiate between PD and ET [2]. Several studies in which imaging was conducted as an outcome measure in patients thought to have early PD found that dopamine function (assessed using DAT SPECT or [^18^F]F-dopa PET) was preserved in approximately 15% of patients [3,4]. This phenomenon, which has become known as Scans Without Evidence of Dopamine Deficiency (SWEDD) is now thought to mostly correlate with dystonic tremor [5]. Such subjects do not show evidence of disease progression on serial DAT studies [6], are not dependent upon dopaminergic medication [7] and careful examination reveals the clinical features to be distinct from PD [8,9].

The more challenging diagnostic consideration is the separation of PD from other akinetic-rigid syndromes. While PD shows a characteristic pattern of impaired dopaminergic function that is asymmetric and affects the posterior more than the anterior striatum [10], this pattern may also be seen in MSA [11] and is therefore insufficient on its own to reliably differentiate between the two conditions. However, MSA is typically associated with loss of dopamine receptors, which are preserved in PD. Thus the combination of pre- and post-synaptic abnormalities of dopamine transmission may help differentiate PD from the Parkinson-plus syndromes [11,12]. Two other radiotracer approaches are worth considering for the differentiation of PD from other akinetic-rigid syndromes. PD is associated with a characteristic pattern of increased glucose metabolism in basal ganglia and cerebellum with concurrent reductions in multiple cortical regions, the so-called PD Related Pattern (PDRP)[13]. This pattern is not seen in MSA, PSP or other akinetic-rigid conditions such as corticobasal syndrome and the pattern of glucose metabolism can be used for diagnostic classification with a high degree of specificity [14,15]. Finally, PD is frequently associated with autonomic dysfunction reflecting degeneration of sympathetic ganglia, whereas in MSA, the degeneration is predominantly central. Thus cardiac sympathetic imaging using [^123^I]MIBG (SPECT), or [^11^C]*m-*hydroxyephedrine or [^18^F]fluorodopamine (PET) is typically abnormal in PD, but preserved MSA and PSP [16-18], although the differentiation may not be entirely reliable [19].

Traditional MRI changes of MSA (increased iron deposition in globus pallidus, rim of putaminal hyperintensity, "hot cross bun sign")[20] and PSP ("hummingbird sign")[21] may not be entirely reliable, particularly in early disease. However, diffusion MRI may allow diagnostic differentiation [22-24], and PSP may be identified by detailed measurements of the midbrain and superior cerebellar peduncles [25]. Multimodal MR techniques assessing diffusivity (microstructural damage), fractional anisotropy (white matter tract integrity) and iron appear to separate PD from controls with a high degree of sensitivity and specificity [26] but have not yet been routinely applied to differential diagnosis.

### Dementia

MRI may clearly be of help in differentiating between Alzheimer disease (AD) and multi-infarct dementia. Amongst the other degenerative causes of dementia, dementia with Lewy bodies (DLB) and frontotemporal dementias are the most important. Structural MRI shows atrophy of the hippocampus and entorhinal cortex in AD [27], as well as involvement of the lateral parietal, posterior superior temporal and medial posterior cingulate cortices. This is in contrast to FTD, where atrophy is more prominent in frontal or temporal poles. However, while the pattern of atrophy may help differentiate some variants of FTD, there is considerable overlap between Pick complex and AD [28]. Newer techniques such as diffusion tensor imaging may be of greater help in the differential diagnosis of degenerative dementias. DTI has demonstrated abnormal white matter in the parietal lobes of patients with DLB compared to AD [29].

Functional studies may be more sensitive in detecting abnormalities that differentiate various forms of dementia. Thus fMRI studies reveal reduced frontal but increased cerebellar activation during performance of a working memory task in FTD compared to AD [30]. In recent years, there has been increasing interest in networks of connectivity that are present during rest. Activity in the so-called default mode network, which includes precuneus, posterior cingulate cortex, orbitofrontal, medial prefrontal and ventral anterior cingulate cortex, as well as inferior parietal, left dorsolateral prefrontal and left parahippocampal gyrus, is suppressed during performance of a cognitive task [31]. Resting state or task-free fMRI identifies networks whose activity is correlated over time. Default mode network activity is reduced in AD compared to controls [32]. In contrast, behavioural variant FTD is associated with increased activity in the default mode network but reduced activity in the so-called salience network, which encompasses fronto-insular, cingulate, striatal, thalamic and brainstem nodes [33]. A detailed discussion of functional network disruption in degenerative dementias has recently appeared [34].

[^18^F]fluorodeoxyglucose (FDG) PET shows reduced glucose metabolism in parietotemporal cortex in AD. FDG PET has a small positive influence on sensitivity and a modest influence on specificity compared with initial clinical evaluation in AD. Positive and negative predictive values for FDG PET compared to the gold standard of pathological diagnosis are both approximately 0.8 and PET significantly enhances the diagnostic accuracy of clinical evaluation [35]. Dementia with Lewy bodies results in a greater degree of occipital hypometabolism than AD and FDG PET may thus assist in the differentiation of the two disorders, as verified at autopsy [36]. Similarly, FDG PET may help improve diagnostic accuracy for FTD versus AD, as the former typically affects frontal lobes, anterior temporal and anterior cingulate cortex [37]. Dementia with Lewy bodies can also be differentiated from AD based on imaging evidence of dopamine deficiency using dopamine transporter SPECT [38] or [^18^F]F-dopa PET [39].

While patterns of glucose hypometabolism may be helpful in distinguishing between AD and control state, as well as between AD and other dementing disorders, in recent years interest has focused on the use of agents that label amyloid. The best known of these is [^11^C] labeled Pittsburgh Compound B (PiB), a thioflavin derivative that appears to be specific for β-amyloid deposition [40]. A number of other [^18^F]-labeled agents have been developed and these may prove useful, particularly for centres at some distance from a cyclotron, given the longer of half-life of [^18^F] (approximately 2 hours) compared with that of [^11^C] (approximately 20 minutes). PiB binding is increased in widespread cortical and subcortical regions in AD. This may clearly be helpful for the differentiation of AD from frontotemporal dementia, although a sizeable minority of FTD patients demonstrate increased PiB uptake and it is not clear whether this may represent concurrent AD pathology [41]. PiB binding may also be present in patients with DLB or PD-dementia, but this appears to reflect binding to β-amyloid rather than to α-synuclein [42-44].

## Early detection of preclinical disease

### Parkinson's disease

[^18^F]F-dopa PET shows evidence of asymptomatic dopamine dysfunction in subjects exposed to the nigral neurotoxin N-methyl-4-phenyl-1,2,3,6-tetrhydropyridine (MPTP)[45] and in unaffected twins of subjects with PD, particularly monozygotic [46]. In monozygotic twins, the rate of decline in dopamine function is greater than normal, and some of these individuals will go on to develop symptomatic disease [46]. Progressive decline of F-dopa uptake is also seen in individuals exposed to MPTP, associated with emergence of new clinical signs [47].

Radiotracer imaging shows evidence of abnormal dopaminergic function in asymptomatic individuals from families with known dominantly inherited PD, such as PD due to mutations in *LRRK2*[48]. Using standard PET measures, reductions in dopamine transporter (DAT) binding appear early, sometimes many years prior to the expected age of onset, whereas the emergence of clinical signs is associated with reduced F-dopa uptake [49]. However, by using longer scan times with F-dopa, one can determine effective dopamine turnover and this appears to be the earliest indicator of abnormalities in *LRRK2 *mutation carriers [50]. Whether abnormal dopamine turnover is simply the earliest measurable reflection of dopamine dysfunction in those destined to develop disease or whether it is directly related to mutated LRRK2 function in the absence of neuronal dysfunction is as yet unclear.

Abnormal dopamine function has also been detected in asymptomatic heterozygous carriers of recessively inherited mutations for PD [51,52]. The significance of this is unclear and scan abnormalities in such individuals progress much more slowly than is the case for idiopathic PD, with no evidence of clinical manifestations over 5 years of follow up [53]. However, asymptomatic heterozygous single mutation carriers for both Parkin and PINK1 have morphometric abnormalities (increased basal ganglia grey matter volume)[54] as well as evidence for motor reorganization when performing a task of internally selected finger movements [55,56].

Hyposmia is a common feature of PD and may be present for some years prior to the manifestation of motor abnormalities. A number of studies have demonstrated abnormalities of DAT binding in hyposmic 1^st ^degree relatives of patients with PD, some of whom then go on to develop PD at follow up [57]. Transcranial sonography reveals increased echogenicity in the substantia nigra of patients with PD, thought to reflect increased iron deposition [58]. Hyposmic individuals who also display abnormal nigral echogenicity have a high likelihood of demonstrating abnormal reductions in DAT binding [59].

REM sleep behaviour disorder (RBD) is associated with the future development of neurodegenerative disease, mostly PD, in more than 50% of subjects over a period of 12 years [60]. Patients with isolated RBD may demonstrate reduced striatal dopamine function as assessed by DTBZ PET [61]. A recent longitudinal study using SPECT found reduced DAT binding at baseline in 50% of subjects, a more rapid rate of decline in DAT binding in RBD subjects compared to controls, and the emergence of PD in 3/20 RBD patients (those with the lowest DAT binding) over the follow-up period of 3 years [62]. RBD has also been associated with abnormal nigral echogenicity [63] and there are recent reports of abnormal fractional anisotropy in the midbrain and pontine tegmentum [64] as well as microstructural changes in the white matter of the substantia nigra [65] of RBD subjects.

### Dementia

In the last decade, there has been growing interest in the use of imaging to determine which subjects with mild cognitive impairment (MCI) may go on to develop AD. Measures of entorhinal cortex, superior temporal sulcus and anterior cingulate cortex were able to discriminate approximately 75% of those subjects with restricted memory impairment from those who converted to AD within 3 years in one study [66]. Volume based morphometry confirms the presence of early atrophy in medial temporal structures including amygdala, anterior hippocampus, entorhinal cortex and fusiform gyrus in amnestic MCI subjects who progress to AD [67], but those who convert from MCI to AD also show signs of atrophy outside the medial temporal lobes, particularly lateral temporal and parietal cortex [68]. The Alzheimer Disease Neuroimaging Initiative follows a large cohort of subjects with multimodal imaging. Grey matter density is reduced in amygdala, hippocampus and insula as well as frontal and temporal cortex of MCI converters compared to MCI with stable cognitive function. Cortical thickness in lateral temporal cortex, inferior parietal gyrus and precuneus also differentiates between the two groups [69].

Diffusion tensor imaging may prove more sensitive than traditional structural MRI measures. A recent study found that amnestic MCI converters had changes similar to those seen in established AD, whereas subjects who did not convert had patterns similar to healthy controls. The two amnestic MCI groups (converters vs. non-converters) were distinguished by mean diffusivity in total grey and white matter, hippocampus, insula, frontal and parietal white matter, occipital grey and white matter, as well as fractional anisotropy of temporal white matter [70].

Early stages of disease may be associated with adaptive changes in an attempt to compensate for the functional deficit. It is therefore of interest that MCI may be associated with increased hippocampal blood flow during performance of a face-name encoding task, although the response is decreased in patients with established AD [71]. Mild cognitive impairment is associated with glucose hypometabolism out of proportion to the degree of atrophy in entorhinal cortex [72]. Entorhinal cortex hypometabolism also predicts cognitive decline in healthy subjects, particularly those who carry the ApoE4 genotype [73,74]. Progression of MCI to AD is predicted by a combination of impaired episodic memory and glucose hypometabolism [75], or by combining multiple biomarkers in the form of anatomical MRI, FDG PET and CSF measures of tau:Aβ[76]. Somewhat more than 50% of MCI patients have evidence of abnormal amyloid deposition on PET [77,78] and this appears to be associated with an increased risk of conversion to AD [79-81].

## Concluding comments

The differential diagnosis of neurodegenerative disorders is largely based on careful clinical assessment, but imaging techniques may provide useful adjunctive information. In the case of PD, radiotracer imaging can identify those who have abnormal dopaminergic function, but relatively specialized approaches are required to differentiate the various conditions that may result in parkinsonism. Standard structural MRI is of relatively limited utility in PD, but newer techniques that assess connectivity and microstructural damage may play a role. In the dementias, volumetric analysis of regional tissue loss may be useful for differential diagnosis, but the specificity is likely to be enhanced when combined with fMRI or FDG PET measures of cerebral activation and connectivity, as well as diffusion tensor measures of anatomical connectivity. In the case of AD, specific imaging markers of abnormal protein deposition are available, but this is not yet the case for the other degenerative dementias or for the parkinsonian conditions. The use of multimodal imaging, especially when combined with other biomarkers, shows increasing promise for the detection of preclinical disease in individuals at increased risk and this may be of enormous utility as better neuroprotective strategies are developed.

## Competing interests

The author declares that they have no competing interests.
